# Bridging pathways: SBP15 regulates GOBLET in modulating tomato axillary bud outgrowth

**DOI:** 10.1093/jxb/erad328

**Published:** 2023-09-12

**Authors:** Rameshwar Sharma, Yellamaraju Sreelakshmi

**Affiliations:** Repository of Tomato Genomics Resources, Department of Plant Sciences, University of Hyderabad, Hyderabad-500046, India; Repository of Tomato Genomics Resources, Department of Plant Sciences, University of Hyderabad, Hyderabad-500046, India

**Keywords:** Apical dominance, auxin, axillary buds, miR156, shoot branching, *SPL/SBP* genes, tomato

## Abstract

This article comments on:

Barrera-Rojas CH, Vicente MH, Brito DAP, Silva EM,Muñoz Lopez A, Ferigolo LF, Carmo RM, Silva CMS, Silva GFF, Correa JPO, Notini MM, Freschi L, Cubas P, Nogueira FTS. 2023. Tomato miR156-targeted *SlSBP15* represses shoot branching by modulating hormone dynamics and interacting with *GOBLET* and *BRANCHED1b*. Journal of Experimental Botany 74, 5124–5139.

**Axillary buds (ABs) are dormant buds located in the leaf axils of plants, which have the potential to develop into branches or flowers under appropriate conditions. At the molecular-genetic level, the miR156/*SPL*/*SPB* module regulates the development of ABs in plants, thus influencing plant architecture. Auxins are plant hormones that regulate various aspects of plant growth and development, including AB activity. Barrera-Rojas *et al*. (2023) show that suppressing AB outgrowth elevates auxin levels and lowers *GOBLET* expression, probably by suppressing its transcription by SBP15. Their findings provide insights into the regulation of AB outgrowth and tomato shoot architecture.**

The influence of the shoot apex on suppressing the growth of lower organs is well known. In orchards, gardeners prune the tips of plants to promote growth in lower areas. However, in plants such as tomatoes, excessive shoot branching diverts resources from fruit development and reduces yield (Pino *et al.,* 2022). Consequently, it is desirable to eliminate or reduce shoot branching in tomatoes to avoid manual pruning of axillary branches. Mutations in tomato, such as *LS* (Schumacher *et al.,* 1999) and *BL* (Schmitz *et al.,* 2002) suppress branching but also negatively affect fruit yield (Groot *et al.,* 1994).

Our knowledge of the mechanisms governing AB formation, quiescence, and outgrowth through the activation of axillary meristems (AMs) is limited. Auxin, a plant hormone, plays a crucial role in regulating AB outgrowth. Application of auxin to a decapitated shoot apex restores apical dominance and inhibits AM activation, establishing auxin as a master regulator of AB growth. Although auxin is transported basipetally in the stem, it does not enter ABs. Inhibition of basipetal auxin transport in the stem by phytotropic agents promotes AB outgrowth (Beveridge *et al.,* 2000; Fichtner *et al.,* 2017). While auxin acts as an inter-organ communicator, a specific gene module regulates the suppression or activation of AB growth. Transcription factors belonging to the TCP family, such as TEOSINTE BRANCHED1 (TB1) or BRANCHED1 (BRC1), negatively regulate AB outgrowth (Liu *et al.,* 2017). In tomato, two paralogs of *BRC1*, *BRC1a* and *BRC1b*, are expressed in dormant ABs and down-regulated upon bud activation. Loss-of-function studies indicate that BRC1b plays a role similar to the ancestral BRC1 gene in suppressing AB growth (Martín-Trillo *et al.,* 2011).

The expression levels of *BRC1* genes are regulated by the SPL/SBP transcription factor family, which is modulated by miR156 (Xie *et al.,* 2020). The *miR156/SBP/SPL* regulatory hub operates through the cleavage or translational repression of *SPL* transcripts by *miR156* (Wang and Wang, 2015). Overexpression of *miR156* in tomato leads to increased shoot branching (Silva *et al.,* 2014) and a reduction in *SPL*/*SBP13* transcripts (Cui *et al.,* 2020). While the *miR156/SBP/SPL* module regulates shoot branching in tomato, the downstream processes are still not fully understood.

## miR156-resistant SBP15 elicits strong quiescence in axillary buds

To investigate the molecular basis of shoot branching, Barrera-Rojas *et al*. (2023) manipulated genetic pathways associated with AM activation in Micro-Tom, a dwarf tomato variety. Consistent with previous reports, Micro-Tom plants overexpressing *miR156* displayed more activated ABs than wild-type plants. This phenotype was not limited to Micro-Tom, as the high shoot branching trait could be transferred to a commercial tomato cultivar—Moneymaker. These results established that *miR156*-targeted genes negatively regulate shoot branching in tomato.

One target of *miR156* is the *SPL/SBP* gene family, whose members act as repressors of shoot branching in other species such as rice (Dai *et al*., 2018) and Arabidopsis (Gao *et al.,* 2018). Barrera-Rojas *et al*. generated a phylogenetic tree comparing SBP-box domains from different species to identify potential targets of *miR156* in regulating tomato branching. Among the 15 *SPL/SBP* genes in tomato, SBP15 grouped with known shoot branching-associated SPL genes. This homology suggested that *SBP15* could be the target of *miR156* for negatively regulating shoot branching in tomato. The expression pattern of *SBP15* transcripts overlapped with the *miR156* expression domain in ABs, further supporting this notion.

To investigate the role of SBP15 in regulating ABs, Barrera-Rojas *et al*. overexpressed an miR156-resistant version of *SBP15* (*rSBP15*) in Micro-Tom. The rSBP15 plants exhibited AB formation, but the buds were smaller and remained at an early/dormant stage. Surprisingly, a gene-edited null mutant of *SBP15* (*sbp15*^*CRISPR*^) did not show noticeable changes in shoot branching compared with wild-type plants, indicating that the down-regulation of *SBP15* by *miR156* was sufficient to initiate ABs in tomato. The phenotypes of *rSBP15* and *sbp15*^*CRISPR*^ plants collectively indicated that the miR156-targeted down-regulation of *SBP15* initiates AB outgrowth in tomato, possibly in conjunction with other SBPs such as SBP13 (Cui *et al.,* 2020), to establish the vegetative architecture of tomato.

## Strong axillary bud quiescence was associated with elevated auxin/abscisic acid levels

The smaller and less developed ABs in *rSBP15* plants compared with those of Micro-Tom suggested that the *miR156/SBP15* pathway might affect the polar auxin transport stream (PATS). By hindering auxin export from ABs, *rSBP15* probably restricted their outgrowth. This view was supported by the decapitation experiment, where the topmost AB in *rSBP15* plants exhibited stronger dominance and grew significantly longer than the lower ABs. Additionally, the application of a PATS inhibitor [1-naphthylphthalamic acid (NPA)] revealed that *rSBP15* ABs were more responsive to the reduction in auxin transport compared with Micro-Tom ABs. These findings suggest that miR156-targeted *SlSBP15* may influence auxin transport in ABs, affecting their activation and growth.

The regulation of auxin synthesis and transport is complex, and its relationship with the *miR156/SBP15* pathway is not well understood. To unravel the underlying genic regulation causing AB arrest, Barrera-Rojas *et al*. analyzed the transcriptome of ABs from Micro-Tom and its transgenic *156-OE* and *rSBP15* plants. Consistent with the quiescence of ABs in *rSBP15* plants, genes related to cell division and protein synthesis were transcriptionally repressed. The transcriptome analysis also revealed that a marker for AB dormancy, *DRM1*, was down-regulated in *156-OE* plants and up-regulated in *rSBP15* ABs. Notably, auxin-associated genes, including *LAX2* and *PIN9* involved in auxin transport, were down-regulated in *rSBP15* ABs, correlating with their growth arrest. The *rSBP15* ABs had higher endogenous indole-3-acetic acid (IAA) levels and accumulated abscisic acid (ABA), in line with their arrested development. An up-regulation of ABA is also observed in arrested ABs of shaded Arabidopsis plants wherein BRC1 activates genes encoding HD-ZIP transcription factors, which trigger a genetic cascade elevating ABA levels by up-regulating *NCED*, an enzyme involved in ABA biosynthesis (González-Grandío *et al.,* 2017).

## SBP15 suppresses *GOBLET* expression in quiescent buds

In species such as wheat, rice, and Arabidopsis, homologs of *SBP15* genes inhibit shoot branching by activating *TB1/BRC1*-like genes. In tomato, *BRC1a* and *BRC1b* are expressed in arrested ABs and down-regulated upon bud activation. Consistent with previous reports that BRC1b is the primary regulator of branching (Martín-Trillo *et al.,* 2011), yeast two-hybrid (Y2H) assays and intensity-based Förster resonance energy transfer (APB-FRET) assays demonstrated that SBP15 interacts with BRC1b but not with BRC1a. Functional analysis revealed that SBP15 and BRC1b cooperatively enhanced the activity of the *NCED* promoter but had opposite effects on the *PIN9* promoter. A direct interaction between SBP15 and BRC1b proteins in tomato is surprising, as in rice, the SBP15 homolog IPA1/SPL14 is an upstream regulator of *OsTB1* expression (Lu *et al.,* 2013). Nonetheless, the above findings suggest that SlSBP15 and SlBRC1b can modulate the expression of common targets in a cooperative or antagonistic manner.

**Fig. 1.**
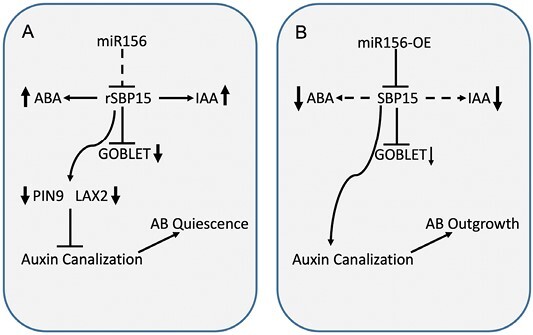
The miR156/SBP15 module controls tomato axillary bud (AB) growth. (A) Overexpression of miR156-resistant SBP (*rSBP15*) strongly induces AB quiescence. This quiescence is characterized by the down-regulation of auxin transporters PIN9 and LAX2, and the accumulation of IAA and ABA. rSBP15 also decreases the expression of *GOBLET*, a leaf dissection-related gene. The down-regulation of auxin transporters may prevent the canalization of auxin, thereby leading to AB quiescence. (B) The overexpression of miR156 down-regulates SBP15 levels, resulting in reduced IAA and ABA levels in the bud. The impact of SBP15 on GOBLET levels in ABs is much less pronounced than in *rSBP* plants. The decrease in IAA levels is likely to be caused by the initiation of auxin canalization, which promotes the outgrowth of ABs.

An intriguing discovery was made regarding the down-regulation of the *miR164*-targeted gene *GOBLET* (*GOB*) in the ABs of both *156-OE* and *rSBP15* plants (Fig. 1). In tomato, *GOB* plays a prominent role in leaf dissection, and its expression is regulated by auxin. The significant reduction in *GOB* transcript levels in *rSBP15* ABs suggested that SBP15 might directly target *GOB*. Co-expression analysis in *Nicotiana benthamiana* leaves confirmed that high levels of *rSBP15* repressed the expression of *GOB* by probably binding to its promoter. Crossing *rSBP15* plants with a *Gob-4d* mutant only partially restored the highly branched phenotype of the mutant, indicating that *rSBP15* had epistatic control over the highly branched *Gob-4d* mutant. These findings establish the reduction of GOB as a novel player in regulating AB outgrowth.

The analysis conducted by Barrera-Rojas *et al*. expands our understanding of the partners involved in the *miR156/SBP*-mediated shoot branching regulation in tomato. Their study highlights the interconnectedness of the tomato *miR156/SBP15* module with auxin, ABA, *GOB*, and the expected *SlBRC1b* gene expression. All these players contribute to the regulatory circuit controlling tomato’s vegetative architecture. The study suggests that maintaining low levels of *GOB* transcripts is crucial for dormant ABs with high *SBP15* expression. However, the SBP15-dependent repression of *GOB* is complex and probably occurs through both auxin-dependent and independent pathways within ABs.

Although the growth of ABs with high *SBP15* expression is impaired, they can still respond to the interruption of polar auxin transport (PAT). This suggests that the development of auxin canalization, which involves specific cell files differentiating into vascular tissues, may be insufficient in smaller ABs with high *SBP15* expression. However, these ABs can grow when the supply of auxin from the shoot apex is disrupted by decapitation.

## What lies in the future?

It is worth noting that several hypotheses have been proposed to explain the release of ABs after decapitation. The prominent one is the ‘auxin canalization’ hypothesis, which states that ABs can only grow once auxin export is initiated. The PIN1 efflux carrier dominantly mediates PAT. It is plausible that PIN9 initiates canalization during early AB activation, followed by the takeover by PIN1. However, the precise roles of different PIN proteins in the onset of auxin canalization await further investigation through *in situ* expression analysis during AB activation. It is worth mentioning that auxin transport can be influenced by various factors. For example, in tomato, the outgrowth of ABs is also a result of the down-regulation of cytokinin levels in plants that overexpress cytokinin oxidase (Pino *et al.,* 2022).

Although auxin plays a central role in all hypotheses related to the onset of AB outgrowth, molecular-genetic studies combined with a systems approach are likely to uncover additional contributing factors. The theory of nutrition diversion, an old concept, suggests that the supply of sugar to ABs activates bud release. The study by Barrera-Rojas *et al*. suggest that ethylene biosynthesis and sugar transport are also modulated in ABs in addition to auxin and ABA. Emerging evidence suggests that sugar transport significantly influences the release of ABs parallel to auxin transport (Beveridge *et al.,* 2023). Similarly, the inhibition of AB growth during shade avoidance strongly correlates with the stimulation of ethylene biosynthesis (González-Grandío *et al.,* 2013).

Our understanding of the quiescence and activation of ABs is still incomplete. However, the work of Barrera-Rojas *et al*. provides new insights into AB growth. The controlled modulation of ABs could have a significant impact on plant architecture and improve agronomic yields.
